# Effects of modifying the World Health Organization standard operating procedures for malaria microscopy to improve surveillance in resource poor settings

**DOI:** 10.1186/1475-2875-13-98

**Published:** 2014-03-15

**Authors:** Sumadhya D Fernando, Ratnasiri L Ihalamulla, Renu Wickremasinghe, Nipun L de Silva, Janani H Thilakarathne, Pandu Wijeyaratne, Risintha G Premaratne

**Affiliations:** 1 Department of Parasitology, Faculty of Medicine, University of Colombo, Colombo, Sri Lanka; 2 Tropical and Environmental Disease and Health Associates (PVT) limited (TEDHA), Colombo, Sri Lanka; 3 Department of Parasitology, Faculty of Medical Sciences, University of Sri Jayawardhenepura, Nugegoda, Sri Lanka; 4 Faculty of Medicine, University of Colombo, Colombo, Sri Lanka; 5 Anti Malaria Campaign, 555/5 Elvitigala Mawatha, Colombo 5, Sri Lanka

**Keywords:** Malaria, Microscopy, Quality of smears, Standard operating procedure

## Abstract

**Background:**

Individuals with fever are screened for malaria in specially-established malaria diagnostic laboratories set up in rural hospitals in the Northern and Eastern Provinces of Sri Lanka. Large numbers of blood smears negative for malaria parasites are being screened daily. Good quality smears are essential to maintain a high diagnostic competency among the technical staff. The modifications made to the World Health Organization (WHO) standard operating procedures to improve the quality of smears have been studied.

**Methods:**

A blinded, controlled, interventional study was conducted in 22 intervention and 21 control malaria diagnostic laboratories. Changes were made to the WHO standard operating procedure protocols to prepare, stain and examine blood smears for malaria parasite detection which were implemented in intervention laboratories. These included wipe-cleaning slides, preparing both thick and thin smears on the same slide, reversing the order of collecting blood for thick and thin smears, dry fixing thick smear for 20–25 minutes under table lamp, polishing the edge of spreader slide with sand paper and fixing the thin smear with methanol if not stained within four hours. Parameters with respect to quality of the smear as per WHO criteria were studied using randomly selected slides, and time taken for the report to be issued was recorded in both groups before and after the intervention.

**Results:**

There were no significant differences observed in the parameters studied at baseline between the two groups or pre and post intervention in the control group. In the intervention group streak formation in thin smears was reduced from 29.4% to 5.0%. The average fixing time of thick smears was reduced from 2.4 hours to 20 minutes. Inappropriate thickness of thick smears reduced from 18.3% to 1.5%. Overall quality of thick smears and thin smears increased from 76.1% to 98.0% and 81.7% to 87.0%, respectively. The quality of slides bearing both thick and thin smears increased from 60.0% to 87.0%.

**Conclusions:**

New protocols with amendments to the WHO standard technical procedures ensure that good quality blood smears are prepared rapidly to diagnose malaria and the time required to issue the reports was reduced.

## Background

Sri Lanka adopted the Global Malaria Control Strategy recommended by the World Health Organization (WHO) in 1994, focusing on early detection and prompt treatment of cases with selected use of vector control methods. With a significant reduction in the number of malaria cases from 264,549 in 2002 to 196 cases in 2007, the country entered the pre-elimination phase for malaria in 2008 utilizing Round 8 funds received from the Global Fund to prevent AIDS, Tuberculosis and Malaria (GFATM) [1]. The strategic plan for phased elimination of malaria from the country 2008–2014, places a higher emphasis on extensive parasitological and entomological surveillance [1]. Following the end of the conflict situation affecting Sri Lanka in May 2009, extensive surveillance activities commenced throughout the country targeting a 100% case detection and laboratory confirmation bringing down the number of indigenous malaria cases to 124 cases in 2011 and a further reduction to 23 in 2012 [personal communication, Dr. Risintha Premaratne, Deputy Director, Anti Malaria Campaign].

Tropical and Environmental Diseases and Health Associates (TEDHA), one of the three principal recipients of the GFATM project, is an implementation partner of the Anti Malaria Campaign (AMC), assisting in malaria surveillance activities. TEDHA has established malaria diagnostic laboratories in 43 rural hospitals located in remote areas of the Eastern Province and Mannar District of the Northern Province with two trained personnel, one a Fever Surveillance Assistant (FSA) to prepare blood smear slides and the other a Parasitology Surveillance Assistant (PSA) to stain and examine the smears under microscope. Fever patients are screened by activated passive case detection in hospital sites and active case detection by conducting mobile malaria clinics in high-risk groups.

Most rural government hospitals in which TEDHA established Malaria Diagnostic Laboratories (MDLs) in January 2010 had poor facilities with six having no water supply, five having no electricity and approximately 27 not having a laboratory sink appropriate for staining. The Standard Operating Procedure (SOP) recommended by the WHO, which has been widely accepted throughout the world, was followed for blood smear preparation and microscopy [2]. However, practical difficulties encountered due to poor resources available at the hospitals, led to a delay in issuing reports causing an additional burden on the patients. With a decline in the number of malaria cases over the past few years, a large number of malaria negative smears are examined at each station (a PSA examines approximately 20–30 slides per day). Technical difficulties in staining and examining blood smears in the MDLs could result in the issue of erroneous reports. The objective of the study was to determine the effects of modifying the WHO standard operating procedures on malaria blood smear preparation and examination.

## Methods

### Changes made to the SOP, introduction of novel methods and laboratory testing of new methodologies

Changes to the SOPs for blood smear preparation and examination and novel techniques that could be introduced were discussed and methods standardized over a period of seven months (January to July 2011) at the Department of Parasitology, Faculty of Medical Sciences, University of Sri Jayewardenepura by the Technical Core Team (TCT) (comprising the two Parasitology Consultants with over 15 years experience in malaria diagnosis and a Technical Resource Person who has over 35 years experience in malaria diagnosis). Items which were required to improve the quality of the smears were ordered and purchased during this time period.

The changes to the SOP and novel techniques which were tested to ensure the quality of slides are as follows.

1. Cleaning of slides prior to smear preparation: the SOP indicates that the slides should be washed with detergents. Microscope slides are imported and generally, the stocks are probably over 12 months old at the time of use. New slides need to be treated in acid-dichromate solution or a detergent [2] and washed before use to remove an oily layer acquired during the manufacturing process. This layer prevents blood smears getting fixed on to the slide properly, thus resulting in smears being dislodged wholly or partially during the wash at the end of staining. In field situations where only basic washing facilities are available, the cleaning process is not practicable. Thus in the field prior to use, new slides are wipe-cleaned with tissue paper until the oily layer is removed completely.

2. Thick and thin blood smears made on different slides: Although the SOP states that the smears should be made on separate slides, this was not possible due to the extra cost of purchasing two slides per person, inadequate storage space for a large quantity of slides and the need to utilize the reagents economically. Thereby at the commencement of operations in January 2010, TEDHA SOPs issued to the laboratories indicated that both the thick and thin blood smears should be prepared on the same slide. However, in some instances the smears extended to touch the two ends of the slide which made it difficult to focus it under the microscope. The techniques introduced ensured that both smears would be 1 cm inner from the edges of the slide and that there would be a gap of 1 cm between the two smears (to prevent methanol flowing on to the thick smear when the thin smear is being wet fixed). This included, (a) writing the label on the outer aspect of the thick smear away from methanol so as to ensure it is not washed out (b) to mark the position where the smears should be prepared on the underside of the slides with a permanent marker so that it will not be affected by methanol. This step helped positioning the two smears correctly avoiding any difficulty in focusing the complete smear under the microscope.

3. Collecting finger prick blood: SOP indicates that a drop of blood for the thin smear is collected first followed by three or four blood drops for the thick smear. The thin blood smear is then prepared to prevent the blood being clotted after exposure to air, followed by the preparation of the thick blood smear [3]. The slides being prepared in the field indicated that the thickness of the thick smears was not sufficient and that the condition of 10–20 WBCs per field in a thick smear which is ideal for parasite detection [2,3] was not being met indicating that the FSAs were not pricking the patient adequately to get sufficient blood for thick smear preparation. With the thick smear being 20 times more sensitive than the thin smear for parasite detection [4] it was of utmost importance to have a good quality thick smear. Thereby, changes were made to the SOP by reversing the order of collecting finger prick blood for thick and thin smears (i.e. colleting the blood for the thick smear first followed by a drop for the thin smear). However, the preparation of the smears i.e. thin followed by thick was carried out based on the SOP. To ensure a thick smear of the appropriate size was prepared, a circle of the size of the thick blood smear was marked on the undersurface of the slides prior to taking finger prick blood.

4. Dry fixing the thick smears prior to staining: the SOP indicates that smears should be kept overnight at room temperature prior to staining so as to prevent the fresh thick blood smear from being washed away at the end of the staining process [2]. However, at the commencement of operations, instructions were given to dry the thick smears at room temperature for approximately two hours, which was sufficient to prevent it being washed away. During the study, the use of a table lamp to dry fix the thick smears was introduced and the ideal time which was required for proper dry fixing of a thick smear so as to prevent it being washed away was standardized to 20–25 minutes.

5. A novel method was introduced to prevent streak formation: The presence of streaks at the tail end of the thin smear, with overlapping red blood cells resulted in a) reduced visibility for detecting a parasite b) reducing the zone of morphology making it difficult to cover the 200 oil immersion fields as required by the SOP [5,6]. This was attributed to the fact that most new microscope slides have rough edges resulting in streak formation. To overcome this, the TCT introduced a novel method by which the edges of the spreader slide were polished with 400 gauge sand paper.

6. Staining of thin smears: The SOP indicates that thin smears ideally should be stained within 4 hours of preparation so that the morphology of the parasites is well preserved [7]. If the slides need to be kept for over 4 hours the TCT introduced fixing the thin smears with methanol thus enabling the slides to be kept till the following day for staining. At the central laboratory, the blood cells showed the crispness seen in a stained fresh smear and the gray/blue background effect caused by plasma was also prevented.

### Selection of laboratories for the intervention

**Figure 1:**
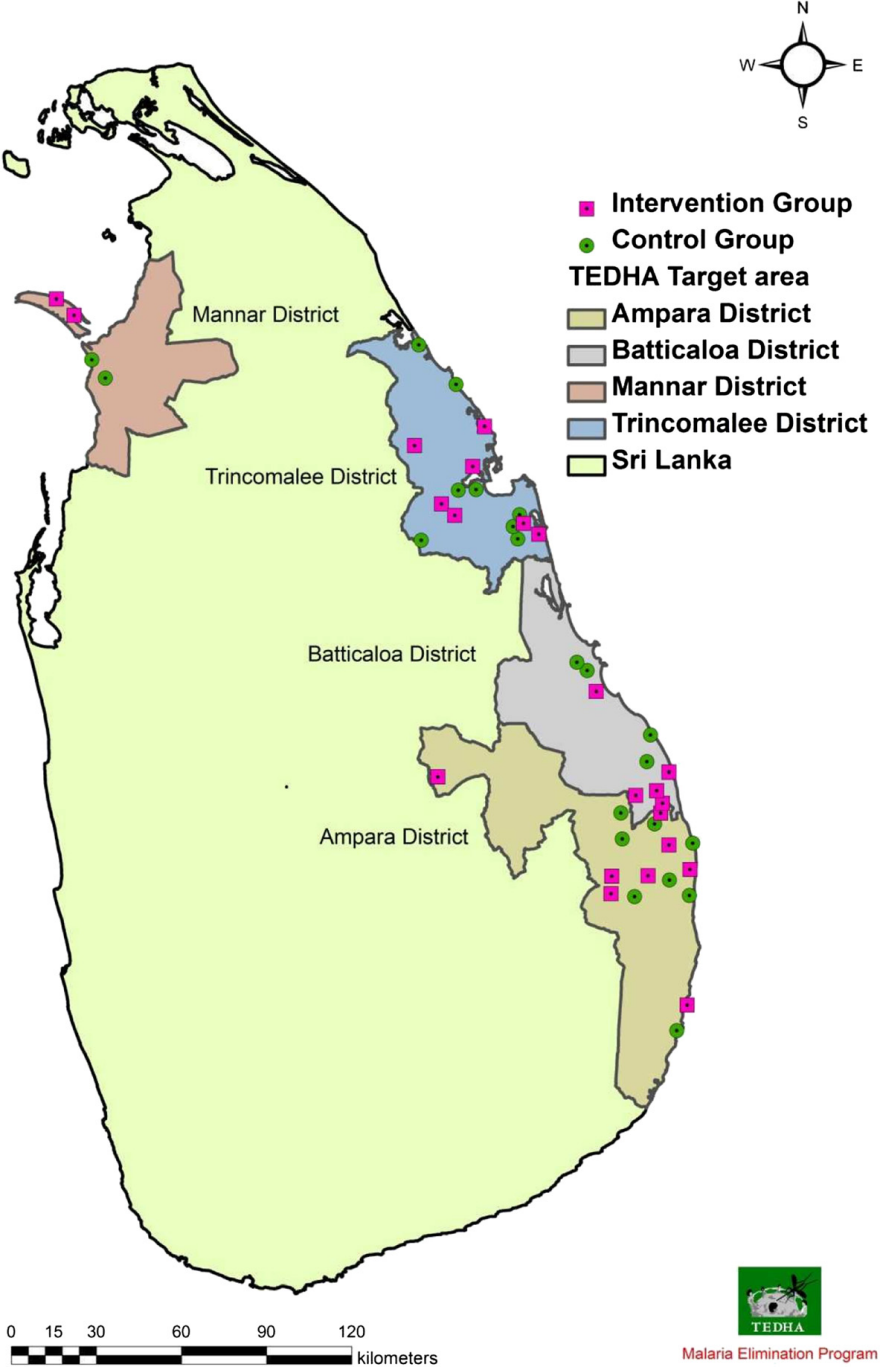
**Distribution of selected malaria diagnostic laboratories.** Forty-three laboratories in four districts were divided into intervention and control groups. There were 22 laboratories in the intervention group which were distributed among the districts as follows, Mannar 2, Trincomalee 7, Batticaloa 5 and Ampara 8. Twenty-one laboratories in control group were distributed as Mannar 2, Trincomalee 8, Batticaloa 4 and Ampara 7.

The study was conducted as a blinded, controlled, interventional study. Forty-three laboratories were given numbers and randomly assigned into intervention and control groups using computer generated random numbers. The control and intervention laboratories in each district are shown in Figure 1. There were 22 laboratories in the intervention group, which implemented the changes to the SOP over a period of two months prior to assessing the quality of slides during the third month and 21 laboratories in control group. The outcome variables studied included quality of the smears, which was assessed using randomly selected slides from the MDLs and time taken to fix the thick smear and issue the report.

### Method of selection of slides for assessment of quality from each laboratory

The slides prepared in each laboratory are packed consecutively in boxes at the end of the week [8]. A Parasitology Surveillance Officer randomly picks two slides from the week’s collection for quality assessment. During the pre and post intervention study periods the same procedure was done under stringent randomization criteria to minimize selection bias. Random selection was done with two papers numbered up to 150 which were known to be the maximum slide number done during a week. If the selected number was beyond the number of slides prepared during that particular week another number was taken. Selected slides were coded, packed and labeled and couriered to the TEDHA Head Office in Colombo where they were de-coded and marked with new coded numbers prior to handing them over to the Technical Resource Person (TRP).

### Defining the quality of thick and thin smear

The quality of slides was assessed by the TRP, who is considered as an expert in this study due to his extensive experience in malaria diagnosis. Based on the WHO quality assurance programme for malaria diagnosis [2] each smear was evaluated as “good smear” or “bad smear” by the TRP (Table 1).

**Table 1:** Parameters considered in evaluating good quality thick and thin smears

**Thick smear**	**Thin smear**
Correct thickness	Not too thick
Properly fixed	Correct degree of staining
Correct size	No staining granules
Correct degree of staining	WBC nuclei red
No staining granules	No staining particles
WBC nuclei red	
No staining particles	

The criterion for the above evaluation is whether malaria parasites could be identified or not if present in the blood smear. A smear was considered of “good quality” if it had the folowing characteristics. Thick smear: a) Hundred microscope fields with a WBC density of 5- > 10 per field could be examined; correct thickness is based on this fact, b) Even if the smear is partially fixed, if the portion which is left on the slide meets the above characteristic, c) The smear is of size that it is sufficient to cover 100 fields, d)Staining is of the correct degree so that the WBC nuclei are red or purple, e) Stain granules and particles are not present in such an amount so as to obscure parasite identification. In the absence of any of the above, a thick smear was considered as of “bad quality”.

Similarly, a thin smear was considered as good quality if a) Two hundred microscope fields each with a density of almost 250 RBCs could be examined, b) Staining is of the correct degree so that the WBC nuclei are red or purple, c) Stain granules and particles are not present in such an amount so as to obscure parasite identification, d) In the absence of any of the above a thin smear was considered as of “bad quality”.

### Provision of appropriate materials and instructions to laboratory staff

Following changes were carried out in both intervention and control groups to further address few of the practical difficulties encountered by the laboratory staff in these field settings.

**Figure 2:**
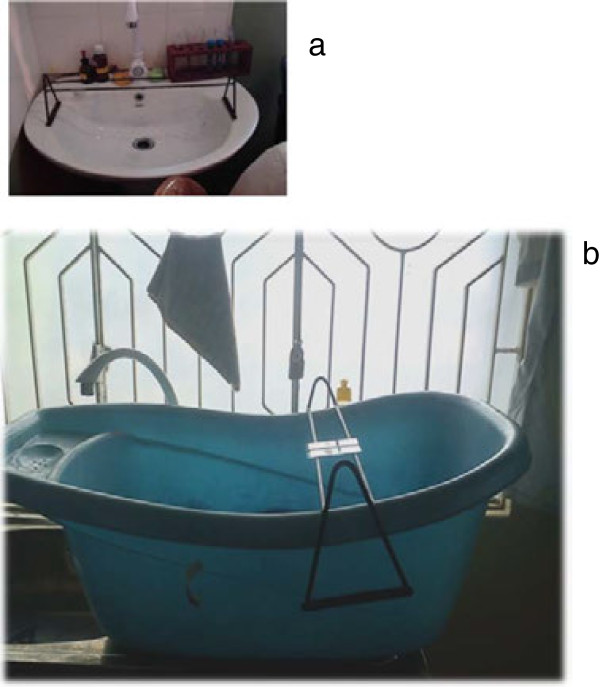
**Alternative staining facilities. a:** Bathroom type small wash basin for staining in some MDLs **b**: Provision of baby bath tubs to all MDLS which did not have appropriate staining facilities. Baby bathtubs provided were beneficial in staining slides. These have sloping sides that allow tilting of staining racks thereby preventing methanol used for fixing thin blood smears flowing onto thick smear. In laboratories where there were bathroom type wash basins (Figure 2a) which are not suitable for staining of slides these bathtubs were quite useful.

1. Obtaining microscopes with mirrors to ensure that reports can be issued without delay in laboratories without electricity supply or during power cuts. Microscopes were purchased prior to the commencement of the operations.

2. Protecting the blood smears from flesh flies and houseflies: Complaints were received from the technical staff that the blood smears were being attacked by flies’ thus creating spaces which made diagnosis a challenge. To prevent this, folding baby cot nets were ordered and purchased by the TCT so as to enable the smears to be covered during dry fixing.

3. Difficulty in staining smears in MDLs with no running water and those which had bathroom type, small wash basins (Figure 2a): The TCT recommended the purchase of baby bath tubs to solve this problem (Figure 2b). The natural curve in the bath tubs on which the staining racks could be placed prevented methanol required for fixing the thin smear flowing on to the thick smear. Prior to adding the stain, the racks could be shifted to the middle where they can be kept horizontal. Using baby bath tubs, also provided the opportunity to place more than one staining rack full of slides for staining, which was thought to be an added advantage in hospitals with a heavy case load, since a large number of smears could be stained at a given time.

Each laboratory was provided with a wall clock of the same brand ordered through the same consignment. Before distribution, the clocks were fitted with new batteries of the same brand and checked to ensure that the time measured were the same in all. The following times were recorded by the FSA and the PSA, (a) time the finger prick blood was taken from the patient (b) time required for dry fixing of thick smears and (c) the time the report was issued by the PSA. Thereby the time required to issue a report was calculated.

### Comparison of the quality of slides at baseline (pre-intervention) and post intervention

A baseline survey of the quality of slides was carried out in all MDLs in September 2011, followed by introduction of the changes to the SOP to the Intervention Laboratories (IL), which was followed over the next two months. Over this period, the Control Laboratories (CL) continued to follow the WHO protocol for blood smear preparation and examination. In December 2011, post-intervention findings regarding the quality of slides were recorded from the MDLs.

### Data entry and analysis

All data were entered into Statistical Package for Social Sciences (SPSS) version 17. Percentage of each studied characteristic was analysed together with significance of difference between groups at 0.05 level using chi-square test. Comparisons were made between the intervention and control at baseline and post-intervention as well as within each group before and after.

## Results

Number of slides examined from the intervention and control laboratories during the months of September and December and the number of slides in which the quality was assessed in the two groups are given in Table 2. All slides were negative for malaria parasites.

**Table 2:** Total number of slides examined and assessed for quality during study period

	**Total number of slides examined for malaria at MDLs**		**Number of slides assessed for quality**	
	**IL**	**CL**	**IL**	**CL**
Pre-intervention period	7322	7665	180	176
(Sept 2011)				
Post intervention period	5850	6330	200	210
(Dec 2011)				

IL: Intervention laboratories.

CL: Control Laboratories.

### Pre-intervention

The quality of blood smears prepared for malaria diagnosis was similar between the smears produced from the ILs and CLs laboratories. Prior to intervention the average time required for dry fixing a thick blood smear was 2.4 hours in the ILs and 2.0 hours in the CLs. Thereby the time required for issuing a report was >2 hours for 66.4% of the smears in the ILs and > 2 hours for 64.4% in the CLs. There were no significant differences between the ILs and CLs with regard to streak formation on the thin smear, the number of thick smears with insufficient thickness or the percentage of slides with good thick smears, thin smears or good thick and thin smears (Table 3).

**Table 3:** Slide characteristics before and after intervention with significance of difference comparing each group

**Characteristic**	**IL**		**CL**		**Significance of differences (p-values)**			
	**Pre (%)**	**Post (%)**	**Pre (%)**	**Post (%)**	**Baseline comparison of two groups**	**Pre and post intervention comparison in CL**	**Pre and post intervention comparison in IL**	**Post intervention comparison between two groups**
Streak formation in thin smear	29.4	5	28.4	26.7	0.83	0.7	<0.001	<0.001
Thick smears with insufficient thickness	18.3	1.5	18.2	17.1	0.97	0.79	<0.001	<0.001
Overall quality of thick smear	76.11	98	75.6	75.2	0.9	0.94	<0.001	<0.001
Overall quality of thin smears	81.67	87	80.7	81.9	0.81	0.76	0.15	0.16
Quality of slides with both thick & thin smears	60	87	61.4	63.3	0.79	0.69	<0.001	<0.001

IL: Intervention laboratories.

CL: Control Laboratories.

### Post-intervention

There were significant differences between the ILs and CLs post intervention indicating an improvement in the quality of slides produced by the ILs. Post intervention, the following characteristics were observed in the smears produced by the ILs which enhanced their quality (a) streak formation in the thin smear reduced (p < 0.001), (b) percentage of thick smears with insufficient thickness reduced (p < 0.001), (c) the percentage of slides with good quality of thick smears improved significantly (p < 0.001) while the quality of thin smears improved, though not statistically significant (p = 0.16), (d) the overall quality of smears being produced (with good thick and thin smears) improved (p < 0.001).

**Figure 3:**
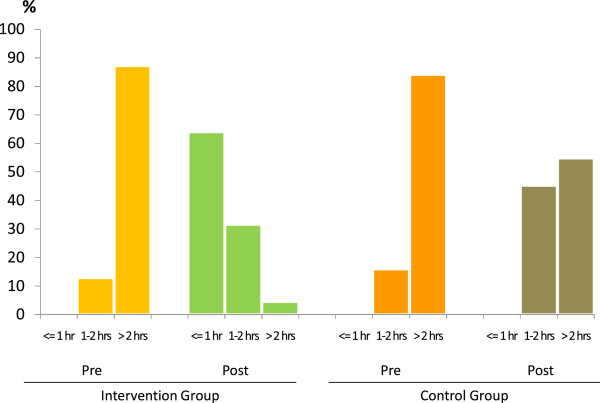
**Reduction in the time taken to issue a report.** Prior to the intervention and post-intervention in the control group none of the reports were issued within one hour due to long dry fixing time of the thick smear at room temperature. Majority of reports were issued more than 2 hours after the collection of samples. But following the introduction of modifications, 64.4% of the reports were issued within one hour in intervention group.

The most significant outcomes post intervention, was that the time required for dry fixing of a thick smear reduced from 2.4 hours to 20 minutes as compared to the CLs where the time required for dry fixing reduced from 2 hours to 1.8 hours. Thus the time required for issuing a report to patients reduced in the ILs, with 31.2% of reports being issued between 1–2 hours and 64.4% being issued in less than an hour (Figure 3).

As expected, significant improvements were seen in the quality of slides in the ILs before and after intervention while there were no significant differences observed in the quality of smears from the CLs before and after intervention (Table 3).

## Discussion

Parasitological surveillance by microscopy is one of the key strategies of the malaria elimination programme in Sri Lanka. The number of reported malaria cases has been on the decline since 2007, with 175 cases (124 indigenous and 51 imported cases) diagnosed during 2011 indicating a slide positivity rate of 0.017%. During this year, TEDHA examined 248,494 blood smears and the AMC 994,546, indicating that a total of 1,243,040 slides were screened for malaria during this year [9]. The quality of the smears is important not only to make a diagnosis but also to identify the species as the treatment modalities used for treatment of *Plasmodium falciparum* is artemisinin combination therapy followed by a single dose of primaquine, while *Plasmodium vivax* is still treated with chloroquine and primaquine at the recommended doses [10].

With extensive surveillance activities being carried out in previous high malaria endemic areas, developing the standards of MDLs, which did not have basic facilities such as running water and electricity so that the technical staff could produce good quality blood smears was recognized as a vital and timely need. This would not only improve the diagnostic capacity but also enable to issue results within a reasonable time to the patients. Despite this necessity, the literature does not provide evidence of any interventions to modify SOPs to address the challenges faced for malaria diagnosis in resource poor settings under field conditions. This publication deals with changes made to the SOPs practiced in internationally accredited laboratories and introduces novel methods used to improve the quality of the smears so as to overcome obstacles due to limited diagnostic facilities. Prior to implementation, changes to the SOP were identified and tested by the TEDHA Technical Core Team at a Central Laboratory. These changes, especially the distribution of table lamps to reduce the time required for dry fixing, when introduced to the field, not only improved the quality of the slides (and thereby the diagnostic ability of the microscopists), but also led to a dramatic reduction in the time required to issue the report.

There is room for bias to affect the results of the methodology adopted in the study although stringent measures were taken to minimize errors. One limitation is the possibility of a higher performance by the field staff in the intervention laboratories due to a greater enthusiasm in the presence of a new modified protocol. Further, information on the changes to the protocol could have filtered informally to the control group resulting in better results post intervention. Nevertheless, the significant improvement that was observed far outweighs the limit that can be explained by the above phenomenon.

Following this study, taking a further step to improve diagnostic skills, all MDLs were issued not only with the new technical SOPs but also with WHO bench aids for malaria diagnosis and a set of reference slides. Strict quality control procedures for preparation of blood smears have been followed by TEDHA since the onset of the programme. To date, two blood smears from each laboratory are checked weekly for quality and feedback given regarding the quality of the smears and methods of improvement. In addition to internal quality control procedures, 10% of the negative slides from each laboratory and all positives slides are sent to the AMC for cross checking and reporting on a weekly basis. The changes to the protocol of SOP, introduction of new procedures and devices have helped to resolve the obstacles to malaria diagnosis under field conditions.

## Conclusions

The changes made to the WHO Standard Operating Procedure could be utilized for Malaria surveillance by light microscopy in malaria diagnostic laboratories situated in all peripheral hospitals of Sri Lanka and could also serve to improve surveillance in other countries facing similar situations.

## Abbreviations

AMC: Anti malaria campaign; FSA: Fever surveillance assistant; GFATM: Global fund to prevent AIDS, Tuberculosis and malaria; MDL: Malaria diagnostic laboratory; PSA: Parasitology surveillance assistant; SOP: Standard operating procedure; TCT: Technical core team; TRP: Technical resource person; TEDHA: Tropical and environmental diseases and health associates private limited; WHO: World Health Organization.

## Competing interests

The authors declare that they have no competing interests.

## Authors’ contributions

SDF designed the study, tested out the procedures and arranged field implementation. RW designed the study and tested out the procedures. RI designed the study, implemented and checked new procedure and examined the slides to assess the quality during the study period, JT ensured the movement of slides between the peripheral hospitals and Colombo Head Office and was responsible for drawing maps. N de S carried out data entry and assisted in analysis. PW designed the study and carried out field implementation. RGP performed statistical analysis. All authors contributed to writing the paper and all authors read and approved the final manuscript.
